# The Effects of Bromine Additives on the Recyclability of Injection Molded Electronic Waste Polymers

**DOI:** 10.1002/gch2.202300157

**Published:** 2023-10-10

**Authors:** Ville Lahtela, Katriina Mielonen, Prashant Parkar, Timo Kärki

**Affiliations:** ^1^ SCI‐MAT Research Platform & Fiber Composite Laboratory School of Energy Systems Lappeenranta‐Lahti University of Technology Yliopistonkatu 34 Lappeenranta FI‐53851 Finland; ^2^ Fiber Composite Laboratory School of Energy Systems Lappeenranta‐Lahti University of Technology Yliopistonkatu 34 Lappeenranta FI‐53851 Finland

**Keywords:** bromine, polymer, recycling, WEEE

## Abstract

Excessive waste amounts, such as waste electrical and electronic equipment (WEEE) and plastic waste, have increased simultaneously with the development of society. Despite the increased material amounts, the recycling rates are too low and those have a great potential to contribute actions toward a circular economy. A certain restricted factor for recycling is the heterogenous nature of materials, such as WEEE‐included additives. This study investigates the effects of a WEEE polymer including bromine on recycling ability, analyzing its physical and mechanical features. The study demonstrates that polymer sorting is profitable for WEEE polymers from the material qualitative perspective, because various processability and material features are achieved in the study between material categories, and especially unidentified polymers have the weakest features in the studied tests. The separation of bromine concentration is also recommended because bromine‐free materials have more advanced features that can be confirmed by statistical analyses. The achieved results support the idea that novel circular economy actions have the potential for effective, efficient WEEE polymer recycling processes with technological innovations, especially when all variables (e.g., recycling cycles and process parameters) are observed and it enables an option to reduce the need for virgin plastic.

## Graphical abstract

The population will increase all the time, as well as the consumption of materials, so the information will contribute to materials’ recycling and reuse. A certain excellent example of materials that need action for reuse are plastic and waste from electrical and electronic equipment (WEEE), and these materials can be physically separated following the principle of sustainable development.

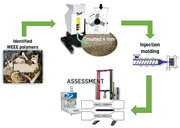

## 1 Introduction

The overconsumption and adequacy of raw materials are global concerns on all sides of the environmental sphere, such as the atmosphere, lithosphere, and hydrosphere, due to increased waste generation. A good example of global economic development is the widely increased consumption of electrical and electronic equipment (EEE), and due to the increased consumption, correspondingly, the amounts of waste electrical and electronic equipment (WEEE) have also increased. For example, the world generated ≈50 Mt of WEEE (e‐scrap) globally, and it is projected will double by 2050^[^ ^1^ ^]^ and almost 75 Mt by 2030.^[^ ^1^, ^2^ ^]^ In 2019, 12 Mt of WEEE was generated in the European Union (EU), corresponding to 16.2 kg per inhabitant^[^ ^2^ ^]^ compared to 11.6 Mt (15.6 kg inh^−1^) in 2014. The European Union (EU) has defined in Directive 2012/19/EU that the waste of electrical or electronic equipment (WEEE) as electrical or electronic equipment (EEE) is waste within the meaning of Article 3 (1) of Directive 2008/98/EC. WEEE includes all components, subassemblies, and consumables that are part of the product at the time of discarding or can only be replaced by the same specifically designed equipment.^[^ ^3^ ^]^

EEE cover applications that require an electric current, battery, or solar energy in order to operate but also other mixture of materials, such as plastics, which are used in EEE for insulation, noise isolation, leak‐proofing, casing, interior structural parts, and electronic components.^[^ ^4^ ^]^ Plastics are inexpensive materials that possess properties such as low thermal and electrical conductivity along with a high strength‐to‐weight ratio, which makes them an ideal choice for use in EEE.^[^ ^5^ ^]^ WEEE plastics are complex systems, including multiple copolymers that are generally immiscible between each other, and different types of polymers can be identified in the plastic content of WEEEs, such as high‐impact polystyrene (HIPS), polycarbonate (PC), polypropylene (PP), poly(styrene‐acrylonitrile) (SAN), and acrylonitrile‐butadiene‐styrene (ABS).^[^ ^6^ ^]^ In WEEE, glass, metal and plastic recovery alone cannot help to meet the targets set by the legislation, as ≈10–30% of WEEE consists of plastics.^[^ ^6^, ^7^, ^8^ ^]^ The latest Organisation for Economic Co‐operation and Development (OECD) report^[^ ^9^ ^]^ indicated that only 9% of plastic waste is recycled, while 22% is mismanaged at the same time when global plastic waste generation more than doubled from 156 Mt in 2000 to 353 Mt in 2019 and globally.^[^ ^9^ ^]^

WEEE plastics include various additives such as chemicals and other materials to improve features of product and production, in which low‐cost filler materials reduce to production costs. Chemical compound additives are generally added to boost the efficiency and aging properties of plastics and are integrated into the plastics used in WEEE. The most common additives used are thermal stabilizers, slip compounds, antistatic agents, pigments, lubricants, light and heat stabilizers, acid scavengers, antioxidants, plasticizers, and flame retardants. Each of these additives plays a discrete role in the properties finally obtained by the plastic.^[^ ^10^ ^]^

WEEE recycling without appropriate actions might add a variety of organic and inorganic pollutants into the ecosystem, such as heavy metals, polycyclic aromatic hydrocarbons (PAHs), polychlorinated biphenyls (PCBs), brominated flame retardants (BFRs), perfluoroalkyl and polyfluoroalkyl substances (PFASs), polychlorinated dibenzo‐p‐dioxins (PCDDs) and polychlorinated dibenzofurans (PCDFs). In addition to the ecosystem, the listed pollutants can adversely affect human health.^[^ ^3^ ^]^ A huge challenge of the reuse of WEEE polymers is its inclusion of hazardous substances, such as flame retardants, which might have a negative impact on the environment and human health without proper treatment. The negative impacts of reuse and recycling will be addressed by pursuing specific legislation, such as WEEE^[^ ^11^ ^]^ and Restriction of Hazardous Substances (RoHS)^[^ ^12^ ^]^ directives. Due to flame retardants, WEEE polymers cannot be recycled via traditional methods if concentration is exceeded. For example, the RoHS limit for bromine concentration is a strict, 0.1% of polybrominated diphenyl ethers (PBDEs). It was previously stated that halogenated compounds (organo‐halogen flame retardants) with chlorinated and brominated agents were included in flame retardants (FRs) with remarkable amounts of global use.^[^ ^13^ ^]^

Brominated flame retardants (BFRs) are an interesting component within WEEE plastics, especially from the viewpoint of circularity.^[^ ^14^, ^15^ ^]^ Several worldwide actions have been made to regulate BFR use and disposal; but still in some countries without regulation in place, informal treatment for valuable materials is still available, causing serious concerns for the environment and the health of the people and communities.^[^ ^13^, ^14^ ^]^ Many studies have identified the presence of BFRs, and the studies demonstrated that color sorting significantly reduces the concentrations of all BFRs.^[^ ^15^, ^16^ ^]^

The aim of this study is to investigate the recyclability of WEEE polymers based on the results of physical and mechanical tests, which were performed for investigated recycled materials. The significance of processing parameters on the properties of recycled polymers will also be discussed, comparing it with corresponding virgin raw materials. The following research questions are highlighted in this research:

1. What is the most efficient combination of WEEE polymers for reuse while maintaining their properties?
2. What is the role of additives in the recycling of WEEE polymers, and what is the significance of bromine in the recycling of WEEE polymers?

## 2 Experimental Section

### 2.1 Materials

Different types of WEEE polymers were used in the investigation and material was obtained from Kuusakoski Oy (Heinola, Finland) that illustrated the typical material composition of WEEE polymers from various batches. The raw material source was based on the WEEE recycling process, which utilized a commercial separation line based on X‐ray fluorescence (XRF) spectroscopy, and a material source containing all types of electric and electronic equipment that was collected from consumer and business sources. The material was industrially sorted into two categories, brominated and bromine‐free polymers, and the main particle size of WEEE polymer was 40–76 mm in diameter.

### 2.2 Methods

**Figure 1:**
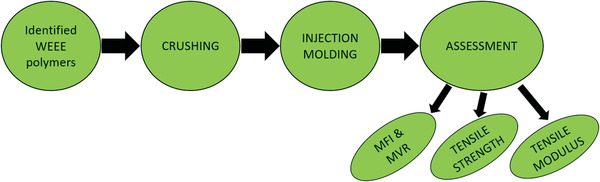
Process diagram of the study.

The material composition of both industrially sorted categories, brominated and bromine‐free polymers, was measured by portable near‐infrared (NIR) spectroscopy equipment (Thermo Scientific microPHAZIR PC, Thermo Fisher Scientific, Waltham, MA, USA) within the spectral range of 1600–2400 nm, reaching information about the major polymer types in WEEE. This study focused on several processing methods, where the recyclability aspects of various WEEE polymers with or without bromine were illustrated. The identified WEEE polymers were processed by crushing and injection molding technologies, and after that, the material features were analyzed by melt flow index (MFI) and tensile property tests. The description of the studied process is illustrated in **Figure** **1**.

#### 2.2.1 Crushing

The polymer materials were granulated after identification. The identified polymers were crushed with a low‐speed granulator apparatus (Shini Plastic Technologies, Inc., New Taipei, Taiwan) at room temperature and 235 rpm for >10 min that was equipped with a 4.00 mm sieve.

#### 2.2.2 Injection Molding

The crushed granulate materials in the study were manufactured by an injection molding process with a BOY 30 (Boy) injection molding machine. In the injection molding process, the materials were heated, melted, and mixed thoroughly with a rotating screw. The melt temperature varied from 190 to 225 °C, the injection pressure was 6.5 MPa, and the injection time was 30–40 s. The injection molding machine has a screw diameter of 24 mm and a mold with a cavity to produce a dumbbell‐shaped tensile test specimen. The 20 samples of each material were manufactured for further analysis, in which the recyclability of the studied WEEE polymers was studied by the results of the melt‐flow index (MFI) and tensile tests.

#### 2.2.3 Melt‐Flow Index (MFI) Test

The melt index of the studied crushed materials was measured with the laboratory melt indexer (Dynisco LMFI‐2NENNNN) according to the standard ISO 1133‐1 method A/B. The MFI test gathered information about the flow rate of the studied materials. The melt flow rate (MFR) and the melt volume flow rate (MVR) were recorded as an average of three replicates for each polymer.

#### 2.2.4 Tensile Test

The reaction of materials to resist force under tension was analyzed by a tensile test (strength and modulus). Mechanical behaviors of the injection molded materials were analyzed from tensile test results with a testing apparatus Zwick Z020 (Zwick Roell group) based on the ISO 527‐2 standard. Testing conditions were congruent for all materials and the test samples were conditioned in a chamber where the temperature and humidity were set to 23 °C and 50%, respectively. Information about the ultimate elongation, tensile strength, and tensile modulus of elasticity was recorded from 20 replicates of each type of material.

#### 2.2.5 Statistical Analysis

Statistical analysis was performed for mechanical results by using Microsoft Excel for the Microsoft 365 MSO program. The correlation coefficient between the tensile and modulus results was analyzed by the “CORREL” function. In addition, Student's *t*‐distribution was calculated for the mechanical results via the “TDIST” function, including one‐ and two‐tailed distribution results. The confidence level was set to 95.0%, indicating that a *p*‐value of 0.05 was used as a significant limit factor. The numeric value (*x*) for distribution evaluation was calculated via Formula (1) as follows:

(1) $$x=\frac{\sqrt{\left(n-2\right)}}{\sqrt{\left(1-r^{2}\right)}}$$

where *n* is the population of the test samples and *r* is the correlation coefficient between the tensile modulus and strength.

## 3 Results

### 3.1 Raw Material Characterization

**Figure 2:**
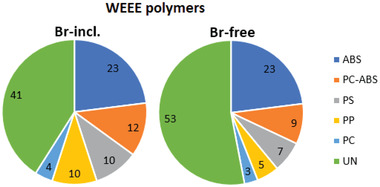
Brominated and bromine‐free WEEE polymers, acrylonitrile‐butadiene‐styrene (ABS), polycarbonate – acrylonitrile‐butadiene‐styrene (PC‐ABS), polystyrene (PS), polypropylene (PP), polycarbonate (PC), and unidentified shares (%) in the material stream.

The WEEE polymers were classified into six categories based on the identified polymer. The five major polymers were ABS, PC‐ABS, PS, PP, and PC, and the sixth category was unidentified polymer (UN), including mainly dark‐color materials. According to the identification of WEEE polymer, material amounts were calculated, and **Figure** **2** presents polymer distribution based on the weight, which was deeply analyzed by energy‐dispersive X‐ray spectroscopy (EDS) in the previous study.^[^ ^17^ ^]^

### 3.2 Recyclability of the WEEE Polymers

After raw material characterization, the four most general material categories (UN, ABS, PC/ABS, PS) were selected for further processing with injection molding and specific material analysis tests, such as melt flow index and mechanical properties. Injection mold parameters (temperature and pressure) were quite restrained, but this way method's further influence can be eliminated because melt temperature and injection pressure cause a significant influence on the product feature.^[^ ^18^ ^]^ The restrained temperature is supported by the conclusion of Franciszak et al.^[^ ^19^ ^]^ that a low processing temperature is a stipulation for recycling plastic scrap. On the other hand, higher temperatures could assist in the processing step because polymers flow better at elevated temperatures.^[^ ^20^ ^]^

**Table** **1** shows the effects of each WEEE polymer on the reprocessing of material, demonstrating the polymers’ mass flow and volume rates. The material category of UN could not be processed in the MFI tester, although the melting temperature varied from 220 to 300 °C. Therefore, the results of the melt flow index from the UN are not available. The results of MVR and MFR correlated clearly with each other, and the deviation of results was minor for each polymer, addressing the high reliability of test results.

**Table 1:** Melt flow index of the studied materials, mass volume rate (MVR), mass flow rate (MFR), and test conditions. The MVR and MFR results were recorded as an average of three replicates. The name of the material consisted of the WEEE polymer and included (Br‐incl.) or excluded (Br‐free) bromine content within the material.

Material	Conditions		Results	
	Load [kg]	Temperature [°C]	MVR [cc/10 min]	MFR [g/10 min]
*ABS / Br‐free*	10	220	20.9	21.8
*ABS / Br‐incl*.	10	220	25.3	26.9
*PC‐ABS / Br‐free*	10	220	23.6	26.5
*PC‐ABS / Br‐incl*.	10	220	16.8	18.6
*PS / Br‐free*	5	200	6.59	5.70
*PS / Br‐incl*.	5	200	5.96	5.69

**Figure 3:**
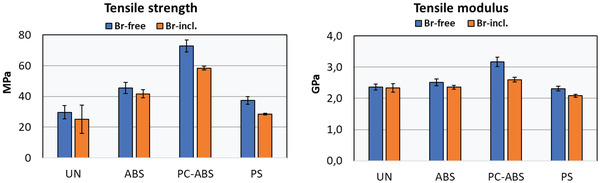
Results of the tensile strength (left) and tensile modulus (right) that was recorded from 20 replicates, with the exception of 18 and 19 replicates for UN‐Br‐incl. and PS‐Br‐incl. samples, correspondingly. The results of bromine‐excluded materials are presented as a blue bar chart with the standard deviations as an error bar, and correspondingly, the results of bromine‐included materials are presented as an orange bar charts with standard deviations as an error bar.

**Figure 4:**
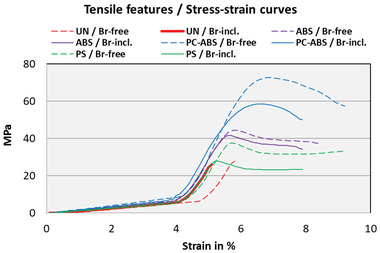
Stress–strain curves of the studied material, as an example, under the tensile property test. Each WEEE polymer is presented in its own color. The results of bromine‐included materials are presented as a continuous solid line, while the results of bromine‐excluded materials are presented as a dashed line.

**Figures** **3** and **4** present the tensile features, which describe the mechanical properties of the reprocessed WEEE polymers. The tensile strength and modulus are presented in Figure 3, separated by the included and excluded bromine results of each polymer. The mechanical properties of bromine‐free materials are presented as a blue bar chart, and corresponding bromine‐containing materials are presented as an orange bar chart, including standard deviations as an error bar. The results of mechanical properties are an average from 20 measurements, but by way of exception, UN‐Br‐contained results consist of 18 measurements, and PS‐Br‐contained results consist of 19 measurements. The smaller measurement amounts of Br‐included UN and PS samples are due to technical errors during the test, but the variation might not be significant.

Figure 4 illustrates typical stress–strain curves of the studied material in the X‐Y scatter chart, to which trend lines have been added using a two‐period moving average for the illustration of stress–strain. Each curve is based on the individual measurement, which corresponds best to the measured average value. A clear observation from the stress–strain curves is that UN materials have little elastic feature because their stress–strain curves stop after the limit value, while the opposite phenomenon is visible with PC‐ABS materials. The samples of PC‐ABS have quite smooth strain curves, demonstrating the elastic feature of the materials. The ABS and PS samples have similar long curves as PC‐ABS, but compared to the PC‐ABS curves, the angle is steeper after the top of the strength value. The steeper angle in the stress–strain demonstrates a minor elastic feature, but still quite good strength, not fragile at least.

Statistical analysis supported the aim of material separation, especially with bromine‐free WEEE polymers. **Table** **2** presents correlation rates between strength and modulus values, and the identified bromine excluded materials have significant correlation results, >0.75 for ABS, PC‐ABS, and PS, while with UN material it was not remarkable. The observation was confirmed by distribution calculations, where the results of the identified values were clearly below the critical level of 0.05. An interesting phenomenon was that this can be verified with bromine‐free polymers, and the results of bromine‐included materials did not confirm this phenomenon.

**Table 2:** Statistical analysis of mechanical features with correlation (*r*) and Student's *t*‐distributions (one‐tailed and two‐tailed).

Material	Strength – Modulus					
	Bromine‐free			Brominated		
	*R*	*p‐*value one‐sided	*p‐*value two‐sided	*r*	*p‐*value one‐sided	*p‐*value two‐sided
UN	−0.0656	0.3918	0.7835	0.0261	0.4590	0.9181
ABS	0.8108	7.3E‐06	1.5E‐05	0.0381	0.4367	0.8734
PC‐ABS	0.7537	6.2E‐05	1.2E‐04	0.3335	0.0754	0.1508
PS	0.8306	2.9E‐06	5.8E‐06	0.3030	0.1036	0.2073

## 4 Discussion

### 4.1 Raw Material Characterization

The study showed that ABS represents the largest volume of a certain identified polymer in the WEEE polymer material stream. Over one‐fifth of all material streams were identified as ABS polymers, and the significance of ABS was even higher when reviewing the second most commonly identified WEEE polymer, PC‐ABS. The typical objects for ABS and PC‐ABS polymers in WEEE have been cathode ray tube (CRT) monitors, printers, copying elements, central processing units (CPUs), and small EEE.^[^ ^21^ ^]^ These two components, PC and ABS, can be easily processed and are also effective for mixtures with flame retardants,^[^ ^22^ ^]^ addressing their widespread use in EEE.

In addition to the flame retardant advantage of ABS, it is an interesting polymer for recycling due to the complex component mixtures. The acrylonitrile phase provides chemical and heat resistance, and the butadiene phase provides mechanical properties for materials.^[^ ^22^, ^23^ ^]^ However, ABS also includes a component of styrene that is considered a possible carcinogen to humans,^[^ ^24^ ^]^ which might restrict its recycling ability due to occupational exposure to styrene. ABS also includes many additives, which is a challenge in effective polymer recycling. According to the study of Vazquez and Barbosa,^[^ ^25^ ^]^ ABS from WEEE includes 8.8 wt% fillers from different natures, such as calcium carbonate, silica, carbon black, talc, and titanium oxide. The role of additives is to develop material features and reduce material and production costs. In particular, calcium carbonate and talc could be utilized at a lower production cost because even 20–40 wt% content addition can retain material performance, depending on the filler properties.^[^ ^19^ ^]^ Overall, numerous additives are added to EEE plastics, including pigments, flame retardants, stabilizers, and plasticizers.^[^ ^21^ ^]^ Additives examples include inter alia, cadmium for pigment, bromine and antimony for flame retardants, or chlorine as a stabilizer, just to name a few.^[^ ^26^ ^]^

However, the largest category was unidentified materials (UN) from the studied WEEE polymers, which is partly explained by the incapability of NIR technology for black polymer classification. The share of dark‐color polymers is congruent with the study of Maris et al.^[^ ^8^ ^]^ where polymer dark color was also 41%. The typical products for dark color additives are televisions and small electronic equipment, which makes plastic identification difficult.^[^ ^21^ ^]^ Previously, black plastic has been an impossible task, e.g., with automated sorters,^[^ ^27^ ^]^ but new capable technologies were innovated for the identification of black plastic, such as midwave infrared (MWIR).^[^ ^28^ ^]^ The color has been a traditional identification parameter for plastic, which is also an interesting aspect for bromine including WEEE polymers because, for example, gray WEEE plastic contains a great amount of bromine, even greater than the RoHS limit.^[^ ^7^ ^]^

### 4.2 Recyclability of the WEEE Polymers

The MVR and MFR values are reported according to the measured amount for mass gram (g) and volume cubic centimeter (cc) at temperatures 200—220 °C with 5.0 and 10.0 kg load. The temperature and load condition numbers were lower with PS polymer, and the same behavior was observed in the results, where PS values were significantly lower compared to the values of ABS and PC‐ABS. The remarkable conclusions of the bromine effect with WEEE polymer cannot be based on the MFI test due to inconsistent results. The MFI results slightly increased with ABS, while they decreased with PC‐ABS and with PS, and the bromine effect was insignificant. In general, the MFI results were quite high compared to the corresponding studies. For example, Tarantili et al.^[^ ^22^ ^]^ investigated WEEE polymers and a mixture of ABS with PC or HIPS showed the highest results of 4.6 and 6.0 g/10 min, respectively. In speculation, the higher MFI results might demonstrate the greater additive amounts with materials in this study. All MFI results were >5 g/10 min, which was discussed as a limit for injection molding with some polymers.^[^ ^29^ ^]^ It must be remembered that material mainly originated from the waste stream with the first recycling cycle, and in the future, material viscosity might increase because it has been found that MFI decreases with the number of injection cycles, indicating high viscosity.^[^ ^30^ ^]^ In the study of Oblak et al.,^[^ ^31^ ^]^ it was stated that significant changes in MFI will occur during the first 30 cycles. Then, with a higher viscosity after various recycling times, the material could also be suitable for other processing methods, such as blow mold or extrusion.

In Figure 3, the best result with PC‐ABS is congruent with previous knowledge that PC has excellent mechanical performance.^[^ ^32^ ^]^ PC and ABS mixtures are often used in EEE due to their effective flame‐retardant properties, and ABS improves the processability of PC due to its elastomeric phase interactions. It has also been found that ABS addition into PC decreases tensile properties, but the optimum composition varies from 10% to 20% in terms of mechanical performance.^[^ ^22^ ^]^ From the mechanical aspect, ABS is a very suitable polymer for recycling because it can maintain mechanical properties, although ABS addition will be increased up to 50 wt.%.^[^ ^22^ ^]^ The tensile strength of the PS material was lower than that of the ABS and PC‐ABS samples, which is explained by its brittle structure, which can be improved for high‐impact polystyrene (HIPS). HIPS is a multiphase system consisting of a rigid phase together with dispersed rubber particles with diameters of 0.5–10 mm.^[^ ^22^ ^]^

It is widely accepted that the properties of polymers deteriorate after reprocessing, due to photooxidation processes or immiscible polymers.^[^ ^33^, ^34^ ^]^ In addition, thermal annealing induces an increase in the crystallinity of recycled polymers, which are lost during processing and correlate with the mechanical properties.^[^ ^35^ ^]^ It can be assumed that the subsequent recycling steps will reduce the mechanical features because it has been stated that multiple recycling passes have a negligible effect on the tensile properties.^[^ ^22^ ^]^ Oblak et al.^[^ ^31^ ^]^ simulated the mechanical recycling of high‐density polyethylene (HDPE), and the results showed degradation of mechanical properties after ten reprocessing cycles. The exact number for reprocessing cyclic is difficult to determine where changes in the properties are visible, but for example, four times reprocessed nylon 12 via injection molding did not cause drastic changes in properties.^[^ ^18^ ^]^ In the study of Lozano‐Gondalez et al.,^[^ ^30^ ^]^ nylon 6 material changes were not remarkable until the eighth cycle, and after the tenth cycle, changes in the properties varied by 10–15% compared to the virgin material. Correspondingly, a 30% decrease in tensile strength was observed after eight recycling steps.^[^ ^36^ ^]^ In turn, the changes can be the opposite because the strength modulus was increased after seven recycling cycles, which can be explained by the increased molecular weight.^[^ ^30^ ^]^ Based on the referenced studies, it could be estimated that the limit for recycling cycles is ≈10 where the changes can be remarkable. In addition to processing parameters and the number of cycles, polymers also influence the features. It was stated that ABS is the prevalent polymer in WEEE, and it also has a significant influence on material recycling and reuse. For example, due to the plasticization effect of ABS into PC, it has great importance on recyclability because minimal exposure to high temperatures was needed, contributing to the avoidance of further decomposition.^[^ ^22^ ^]^

As previously mentioned, processing parameters were restrained so that a significant effect could be avoided in the end‐products. The parameter influence is difficult to forecast because it might be illogical. For example, the tensile strength of the molded product was decreased by an increase in injection pressure, but in contrast, the impact strength was increased with an increase in injection pressure.^[^ ^18^ ^]^ A certain solution to contribute recycled materials could be coinjection molding, where recycled content in the core layer can lead to improvements in some performance categories.^[^ ^37^ ^]^ Therefore, the recycled content could be higher in the core layer, while the skin layer could be a more homogeneous material.

Based on the study by Solis and Silveira,^[^ ^38^ ^]^ the following four general categories for plastic recycling are identified: primary, secondary, tertiary, and quaternary recycling. The same categories are known as WEEE plastic recycling.^[^ ^39^ ^]^ Primary recycling is known as closed‐loop recycling, where the material is mechanically reprocessed, producing a product with equivalent quality, but it cannot process contaminated mixed plastics. Secondary recycling is so‐called mechanical recycling that grinds material into small particles, which are reprocessed into new products. Despite usually including separation and sorting steps in the process, mechanical recycling often downgrades material features. Tertiary recycling means chemical recycling that breaks down polymers into monomers, and quaternary recycling is known as waste incineration for energy recovery.^[^ ^38^ ^]^ In addition to the general categories, other technologies can be applicable, such as advanced thermal chemical recycling methods,^[^ ^40^ ^]^ as well as future technologies, such as “microfactories”.^[^ ^39^ ^]^

As previously mentioned, secondary recycling deteriorates the material properties of recycled materials. For example, Merrild et al.^[^ ^41^ ^]^ showed that mechanical recycling results in 10% material and quality losses with the plastic fraction. Despite the downgraded material feature, mechanical recycling is a cost‐effective recycling method and the most sustainable recovery strategy with a low carbon footprint.^[^ ^36^, ^37^ ^]^ Plastic recycling processes can reduce the need for virgin plastics and save energy costs because recycled plastics use ≈88% less energy than virgin plastics.^[^ ^18^ ^]^ In addition to the economic and energy advantages, other factors must be reviewed in the whole recycling process of WEEE polymers. Additional socioeconomic and sustainability issues also exist, such as increased employment or improved logistics. For example, small or rarely populated countries face the problems of low volume, high transport and infrastructure costs.^[^ ^42^ ^]^

## 5 Conclusion

This paper investigated the significance of bromine in WEEE polymers, especially as a part of the mechanical recycling case. Overall, the plastic amount increases, and more attention must be paid to its recycling because some additives within polymers might cause worries about recycling. Additives have been used for improving polymer features, such as fire retardancy, wherein bromine and its various derivates have been used as replacement fire retardants when the expertise of harmfulness has increased about the traditional fire retardants. Therefore, it can be recommended that bromine‐laden WEEE polymers should be sorted out from the recycling material stream, to achieve better polymer features from the recycled raw materials. In addition, it should be remembered that several other additives also affect the recycling of WEEE polymers, such as mineral fillers, and it must be also considered in the same context, for example in future studies.

This study paper confirms that dark material is a challenge during polymer recycling, but material separation and sorting are profitable because material features are used to identify materials when unidentified materials have a brittle mechanical feature. The melt flow analysis shows that the studied materials are suitable for injection molding treatment, which supports the previous observation that mechanical recycling is an economical management technology for waste plastic. In addition, mechanical analysis shows that bromine‐free materials have better mechanical features than materials with bromine. The achieved observation was confirmed by statistical analyses, which will enable a more valuable recycling stream.

## Conflict of Interest

The authors declare no conflict of interest.

## Data Availability

The data that support the findings of this study are available from the corresponding author upon reasonable request.
